# *Anopheles* larval habitats seasonality and environmental factors affecting larval abundance and distribution in Arjo-Didessa sugar cane plantation, Ethiopia

**DOI:** 10.1186/s12936-023-04782-1

**Published:** 2023-11-15

**Authors:** Arega Tsegaye, Assalif Demissew, Dawit Hawaria, Ashenafi Abossie, Hallelujah Getachew, Kassahun Habtamu, Teshome Degefa, Xiaoming Wang, Ming-Chieh Lee, Guofa Zhou, Delenasaw Yewhalaw, Guiyun Yan

**Affiliations:** 1 College of Natural Science, Department of Biology, Jimma University, Jimma, Ethiopia; 2 School of Medical Laboratory Sciences, Faculty of Health Sciences, Jimma University, Jimma, Ethiopia; 3 Department of Medical Laboratory Sciences, College of Medicine and Health Sciences, Ambo University, Ambo, Ethiopia; 4 Aklilu Lemma Institute of Pathobiology, Addis Ababa University, Addis Ababa, Ethiopia; 5 School of Public Health, Hawassa University, Hawassa, Ethiopia; 6 Department of Medical Laboratory Sciences, College of Medicine and Health Sciences, Arbaminch University, Arbaminch, Ethiopia; 7 Department of Medical Laboratory Sciences, College of Health Sciences, Arbaminch, Ethiopia; 8 Department of Medical Laboratory Sciences, Menelik II College of Medicine and Health Science, Kotebe University of Education, Addis Ababa, Ethiopia; 9 Department of Microbial, Cellular & Molecular Biology, Addis Ababa University, Addis Ababa, Ethiopia; 10 Program in Public Health, University of California at Irvine, Irvine, CA 92697 USA; 11 Tropical and Infectious Diseases Research Center (TIDRC), Jimma University, Jimma, Ethiopia

**Keywords:** Larval, Physico-chemical, Habitat, Seasonality

## Abstract

**Background:**

Water resource development projects are essential for increasing agricultural productivity and ensuring food security. However, these activities require the modification of pre-existing environmental settings, which may alter mosquito larval habitat availability and seasonality. The intensive utilization of current adult vector control tools results in insecticide resistance among the main vectors. When coupled with behavioural resistances, a shift in malaria vector feeding and resting behaviours could compromise the effectiveness of the current adult vector control strategies. Thus, it is important to look for new or alternative vector control interventions for immatures to complement adult control by focusing on different larval habitats and their seasonal availability. Thus, this study investigated larval habitat seasonality and seasonal larval abundance and distribution in irrigated sugar cane plantation settings in Ethiopia.

**Methods:**

*Anopheles* mosquito larval habitats were surveyed and visited twice a month for a period of 14 months. *Anopheline* larvae and pupae were collected, reared, and fed finely ground fish food. Adults were provided with sucrose solution and kept under standard conditions. Female *Anopheles* mosquitoes were identified morphologically and using a species-specific PCR assay. Environmental parameters, which include habitats’ physico-chemical characteristics, were assessed. Larval habitat diversity and larval abundance and distribution were determined across different seasons.

**Results:**

The study revealed that *Anopheles gambiae* sensu lato (s.l.) was the most predominant 4197(57%) vector species, followed by *Anopheles coustani* complex 2388 (32.8%). Molecular analysis of sub-samples of *An. gambiae* s.l. resulted in *Anopheles arabiensis* (77.9%) and *Anopheles amharicus* (21.5%), and the remaining 1.1% (n = 7) sub-samples were not amplified. Physico-chemical parameters such as temperature (t = 2.22, p = 0.028), conductivity (t = 3.21, p = 0.002), dissolved oxygen (t = 7.96, p = 0.001), nitrate ion (t = 2.51, p = 0.013), and ammonium ion (t = 2.26, p = 0.025) showed a significant and direct association with mosquito larval abundance. Furthermore, mosquito larval abundance was correlated with distance to the nearest houses (r = − 0.42, p = 0.001), exposure to sunlight (r = 0.34, p = 0.001), during long and short rainy season animal hoof prints, truck tires/road puddles and rain pools were negatively correlated (r = − 0.22, p = 0.01) and types of habitat (r = − 0.20, p = 0.01). Significant habitat type productivity were observed in man-made pools (t = 3.881, P = 0.01163), rain pools, animal hoof prints, (t = − 4.332, P = 0.00749 in both short and long rainy season, whereas, during dry seasons habitat type productivity almost similar and have no significance difference.

**Conclusion:**

The study found that different larval habitats had variable productivity in different seasons, and that physical and physicochemical features like ammonium and nitrate, as well as the distance between larval habitats and households, are related to larval production. As a result, vector control should take into account the seasonality of *Anopheles* larval habitat as well as the impact of pesticide application on larval source management.

**Supplementary Information:**

The online version contains supplementary material available at 10.1186/s12936-023-04782-1.

## Background

A rapidly growing human population needs to increase agricultural productivity to ensure food security. This requires environmental modifications to get additional land to promote economic growth and alleviate poverty in the developing world [1]. Environmental modification, such as irrigation projects, may alter the existing ecological setting, increase diversity and number of mosquito-breeding habitats [2, 3], and expose larval habitats to sunlight, which in turn increases aquatic temperature and shortens the developmental cycle of larval stages of malaria vectors [4]. It also changes the microclimate, which affects the survival of both immature stages and adult mosquitoes [5–7]. However, past experience indicates less attention is being given to environmental modification problems related to vector-borne disease prevalence, distribution, and public health challenges [8].

In recent years, the prevalence and incidence of malaria have shown significant reductions as a result of the scaling up of vector control interventions and active case management [9, 10]. Though the current vector control interventions focus on adult vectors, mainly the application of indoor residual insecticide spraying (IRS) and insecticide-treated nets (ITNs) [11]. The major malaria vectors have developed resistance to most of the insecticide classes used and potentially to be used for public health [12, 13], which makes the existing vector control approach insufficient for malaria elimination. Hence, the implementation of integrated vector management interventions that focus on the immature stages might become important.

The abundance of immature stages of malaria vectors has been found to increase after rainy seasons and throughout the year in irrigated areas in different parts of Africa [14, 15]. Studies from different parts of the world show that larval control reduces on malaria cases and reduces human vector contact [16–19]. A similar study conducted in western Kenya indicates that the combination of LSM and ITNs results in enhanced protection [20].

Even though larval source management (LSM) has been shown to be effective in different settings in malaria-endemic regions when habitats are few, fixed, and findable [21], numerous aquatic habitats and mosquito larvae species can be found all year in environmentally modified areas [22]. Understanding habitat seasonality and *Anopheles* larvae abundance has a significant impact on designing and implementing possible larval stage control timing and approaches [5, 14, 23]. However, larval abundance may not imply vector productivity in particular habitats; rather, it may be a good predictor of vector availability in the area [24].

Various chemical properties of the larval habitat, such as pH, optimum temperature, and ammonia, nitrate, and sulfate concentrations, have been discovered to affect larval development and survival [25, 26]. Moreover, the physicochemical parameters of the irrigation site are critical for mosquito breeding. In the process of increasing crop production, herbicides, insecticides, fungicides, nematicides, and fertilizers were used in higher quantities [27–29]. Studies indicate that one of the causes of insecticide resistance in malaria vector populations is the extensive use of agrochemicals [30]. Furthermore, the irrigation site may result in rapid larval development due to high proportions of sulfate, nitrate, and phosphate and dissolved solids from the fertilizer, and most of the irrigation system uses river water [31]. River waters contain chemicals such as calcium, magnesium, sulfate, nitrate, phosphate, and dissolved solids in high proportions as nutrients. This might result in the larvae’s exposure to important nutrients for their rapid development and exposure to different chemicals that have similar insecticide groups, which might lead to the development of insecticide resistance in a population through time in generations.

Recent widespread irrigation projects in Ethiopia have increased agricultural productivity and promote economic growth. Such large-scale water resource development projects may increase the burden of malaria by increasing vector density. The risk of such types of activities has not been well studied in Ethiopia. It is important to determine the physical and psycho-chemical properties of breeding habitats, as well as the seasonal abundance of *Anopheles*, in each eco-epidemiological context. In order to develop and implement larval source management methods together with the current intervention strategies, it is essential to have an understanding of the physical and psycho-chemical properties of larval habitats. Despite the fact that several larval ecology studies have been conducted in Ethiopia, there is limited data on the seasonality, succession, and physiochemical properties of larval habitats in relation to larval abundance. Although adult vector control plays a greater role in malaria prevalence and incidence reduction, interventions often focus on adult vectors only or neglect the larval/immature stages.

Thus, this study aimed at understanding the abundance of immature stages of *Anopheline* mosquitoes in irrigated sugar cane development plantations in different seasons and determining the psycho-chemical characteristics of mosquito larval habitats.

## Methods

### Study area

**Fig. 1:**
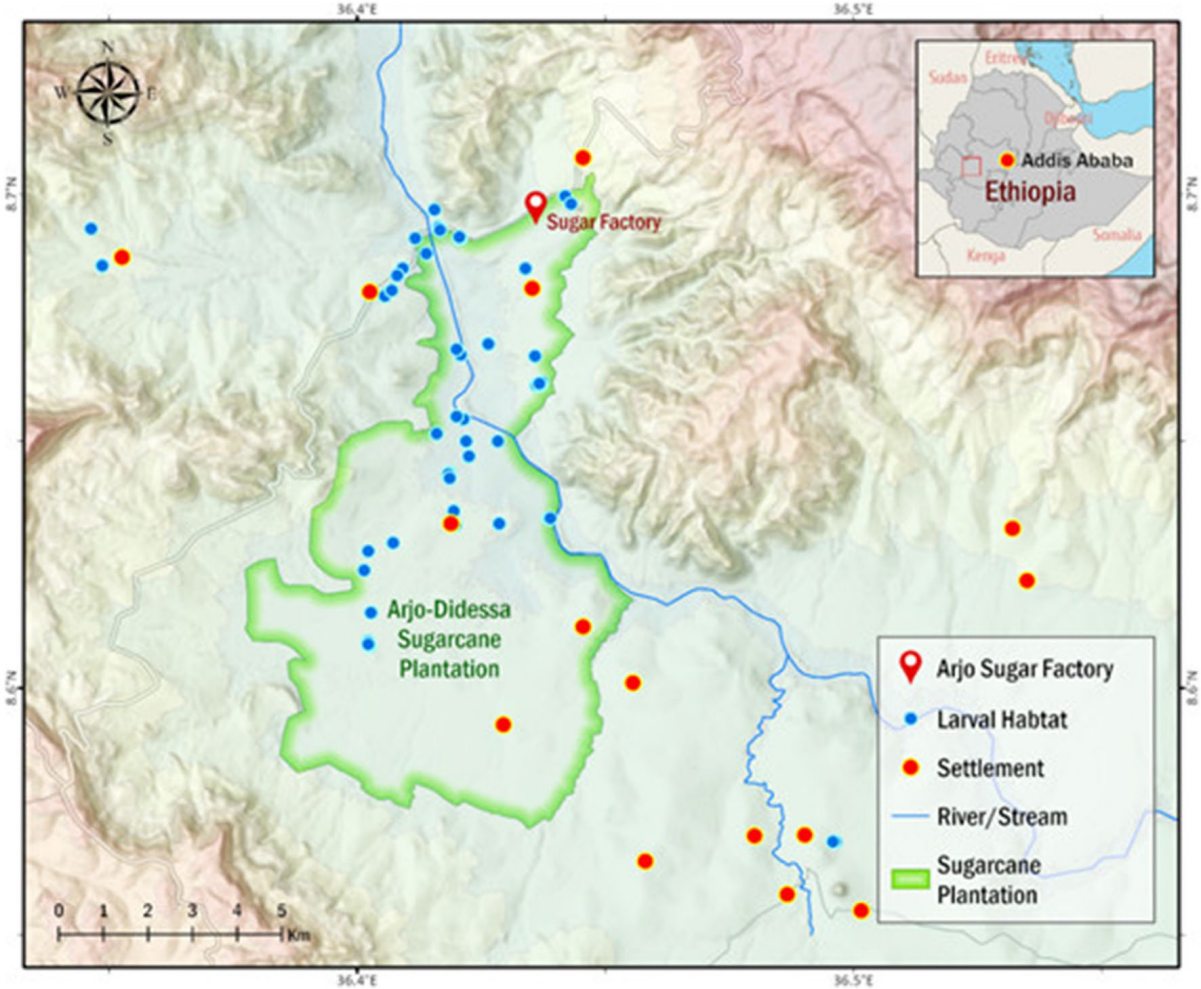
Anopheles larval habitats, Arjo-Didessa sugarcane irrigation area and surrounding areas, southwestern Ethiopia, September 2019–October 2020. This map was created with ESRI ArcGIS Pro 2.8 and publicly available datasets from NASA, OpenStreetMap, and field surveys

The study was conducted at the Arjo-Didessa sugarcane plantation irrigation scheme, which is located between East Wollega and Buno Bedele Zone, a low malaria transmission setting in south-west Ethiopia (8° 41′ 60″ N, 36° 23′ 60″ E) (Fig. 1). The average altitude in the area is 1350 m above sea level. On average, it receives 1400 mm of rain per year [32]. The area mostly has black soil and occasionally has red and brown soil, which has a slow rate of percolation. As result, rainwater can accumulate in the top layers of the soil and form swamps in the area. The area has seasonal malaria transmission, with *Plasmodium falciparum* and *Plasmodium vivax* being the principal malaria parasite species responsible for the majority of the infections: The predominant species in the study area are *Anopheles arabiensis*, *Anopheles amharicus*, *Anopheles pharoensis*, *Anopheles coustani* complex, *Anopheles funestus* group, and *Anopheles squamosus* [15, 33].

### Habitat selection and study site mapping

Identification and follow-up of *Anopheles* larval habitats positive for aquatic mosquito immature stages was conducted from September 2020 to October 2021. First, an exhaustive survey for potential *Anopheline* larvae habitats was done in the study area. A larval habitat was selected for inclusion in the study if it was positive for *Anopheline* larvae during the first survey. Each selected habitat was given a permanent identification number using a wooden frame or stand and was geo-referenced using a handheld Global Positioning System (GPS) unit.

### Larval sampling and identification

Mosquito larval habitats positive for *Anopheles* larvae were surveyed and visited twice a month for a period of 14 month*s* (29 visits). Larval sampling was done using a standard dipper (350 ml, Bio Quip Products, Inc., California, USA). Mosquito larvae habitat physical characteristics like distance to the nearest house, exposure to sunlight, and habitat permanence were measured with visual inspection. Mosquito larvae were immediately filtered using mesh in the field so as to avoid larvae competitors, predators, and unwanted organic debris, and then transported to the field insectary along with water samples taken from the mosquito’s natural breeding sites and reared to adult stages with fish food. The species were sorted by genus and sex during the adult stage following identification keys by Gillies and Coetzee [34, 35] and kept separately in separate cages with a 10% sucrose solution.

### Molecular identification of *An. gambiae* sensu lato

Sub-samples of *Anopheles gambiae* s.l. that emerged from larval collections from the three seasons were randomly selected and identified as species using a species-specific polymerase chain reaction (PCR) assay. In brief, genomic DNA was extracted using a DNA extraction kit (Qiagen, Sigma Aldrich, USA) from whole mosquitoes. PCR amplification was carried out according to the methods of Scott et al. [36] using species-specific primers for *An*. *arabiensis* (AR: 5′-AAGTGTCCTTCTCCATCCTA-3′) and *An*. *amharicus*, formerly *Anopheles quadriannulatus* B (QD: 5′-CAGACCAAGAGAGATGGTTAGTAT-3′). *Anopheles gambiae* (GA: 5′-CTGGTTTGGTCGGCACGTTT-3′) and a universal primer (UN: 5′-GTGTGCCCCTTCCTC GATGT-3′). Then the amplicon was loaded on a 2% agarose gel stained with ethidium bromide and run for gel electrophoresis. *Anopheles arabiensis* from the Sekoru insectary colony and previously confirmed *An*. *amharicus* [33] were used as positive controls.

### Physico-chemical characterization of breeding habitats

Physico-chemical variables such as pH, total dissolved solids, temperature, conductivity, dissolved oxygen, nitrate, ammonium, and orthophosphate ion content of water were measured for all habitats. pH, TDS, temperature, electrical conductivity, and dissolved oxygen were measured using a portable multi-meter (pH Tester 10, Oaklon, USA). The rest, ammonium (NH_4_–N), soluble reactive phosphorous (SRP), and nitrate (NO_3_–N), were analysed according to the standard methods described by APHA [37].

### Statistical analysis

Data analysis was done using the R statistical software package, version 4.2.0. The differences in larval abundance across seasons, habitat types, habitat permanence, distance to the nearest house, and exposure to sunlight were compared using multiple regression analysis. Correlation analysis was used to investigate the relationship between physical and physico-chemical characteristics and *Anopheles* larval abundance. The ratio of the total number of *Anopheles* larvae to the total number of dips taken from each larval habitat was used to calculate the mean *Anopheles* larval density [38].

A mathematical formula was employed to assess the species diversity index, species evenness, and abundance in a community. The species diversity index (SDI) was calculated using Simpson’s Diversity Index Equation [39–41] for measuring species heterogeneity or homogeneity for all weeks in different habitat types. Values near zero correspond to a highly diverse or heterogeneous community, and values near one correspond to a more homogeneous community.

$$D=1-\sum_{I=1}^{N}{Pi^2},$$

where Pi is the fraction of a species that belongs to the nth species, that is, 0 ≤ D ≤ 1 where p is the proportion of individuals in each species, N is the number of species.

Pielou’s evenness index expresses how evenly the individuals in a community are distributed among the different species. Values near one represent a community with near perfect evenness, and they decrease to zero as the relative abundances of the species diverge from evenness [41, 42].

$$J=\text{H}/\text{Ln}(\text{S}),$$

where J′ is Evenness index, H′ is Shannon winner index and used the formula one and S is species richness.

## Results

### Seasonal larval habitat abundance

A total of 78 larval habitats were positive for *Anopheles* during the habitat survey and categorized into eleven types of breeding habitats. In total, 11,031 larvae of *Anopheles* mosquitoes were collected from gate valve leakages, earth-bottom irrigation canals, man-made pools, animal hoof prints, truck tires/road puddles, rain pools, river edges, hippo trenches, spring seepage, farm ditches, and swamps. Of the total collected larvae, 6781 (61.5%) emerged female *Anopheles* species and 2878 (26%) as males, while the remaining 1372 (12.4%) were dead before emerging adults.

Overall, animal hoof prints, rain pools, tire truks/road puddles, and man-made pools yielded a relatively higher larval density, with mean densities of 7.13, 7.05, 6.15, and 5.4 larvae per dip, respectively. The remaining seven breeding habitats, gate valve leakage, earth bottom irrigation canals, river edges, hippo trenches and swamps, farm ditches, and spring seepage, had a mean larval density of 3.13, 3.09, 1.34, 2.25, 1.55, 2.4, and 2.9 larvae/dip, respectively Additional file 1).

**Fig. 2:**
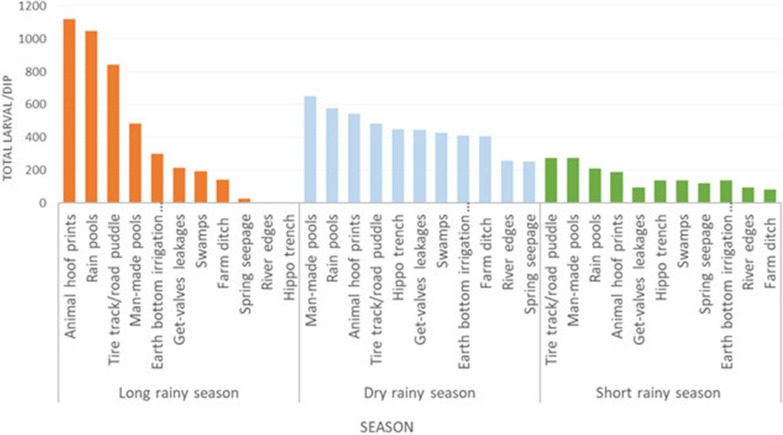
Mosquito larvae abundance by season in various larval habitats

*Anopheles* larvae were more abundant from September to November and declined in December and January. In the long rainy season (June–August), there was high rain overflow/runoff, which reduced mosquito larval abundance. However, during the short rainy season, the larval abundance was relatively similar in different larval breeding habitats. In general, different larvae habitats have different contribution in different seasons, and this differs significantly between seasons (F = 8.2687 p = 0.005) (Fig. 2).

Figure 2 shows that *Anopheles* mosquito abundance was different by season and types of larval habitats. During the long rainy season, rain pools, animal hoof prints, tire tracks/road puddles, and man-made pools contributed in higher proportions, while river edges, hippo trenches, and spring seepage had no contribution. Whereas during the dry season, man-made pools, rain pools, animal hoof prints, gate valve leakage, earth-bottom irrigation canals, hippo trenches, and swamps contributed more, whereas, spring seepage, and river edges contributed a small proportion. During the short rainy season, the contributions of the different larval breeding habitats were relatively similar.

**Fig. 3:**
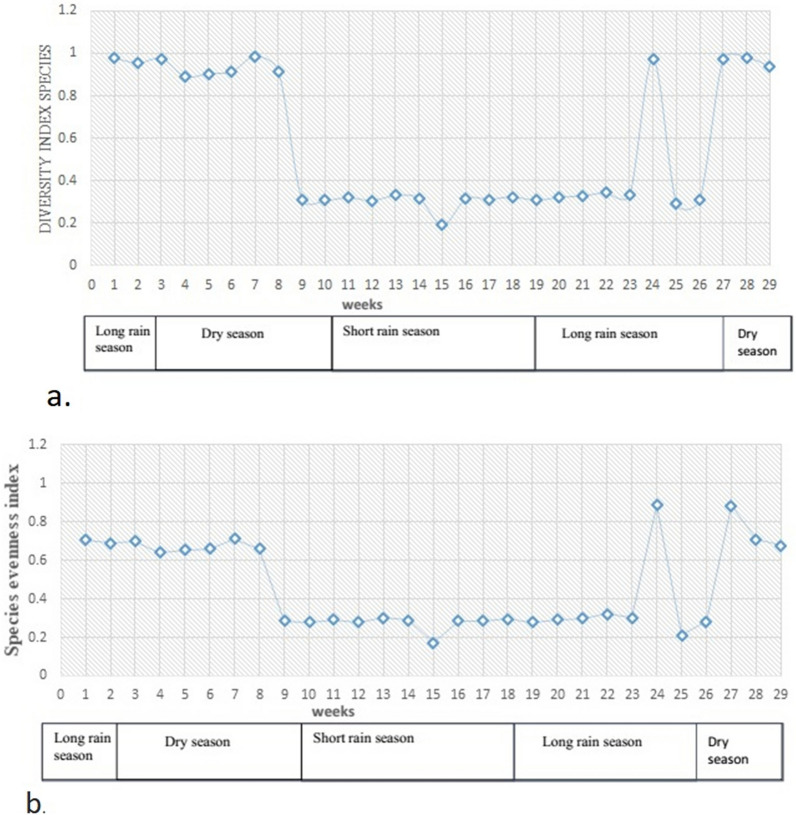
Anopheles species diversity index (**a**) and species evenness index (**b**) in Arjo-Didessa sugar cane plantaton, Ethiopia

Over the study period, the Simpson model showed that there were variations in species diversity over the sampling weeks (Fig. 3a) and seasons: the dry season had a Diversity Index (DI) of 0.95, the short rain season (DI = 0.3), and the long rain season (DI = 1.0). Whereas low species diversity was found between larval habitats; DI was between 0.963 and 0.67. The pielous evenness model (J) indicates that species were distributed unevenly throughout the season in different habitats (Fig. 3b).

### *Anopheles* species abundance and species composition

**Fig. 4:**
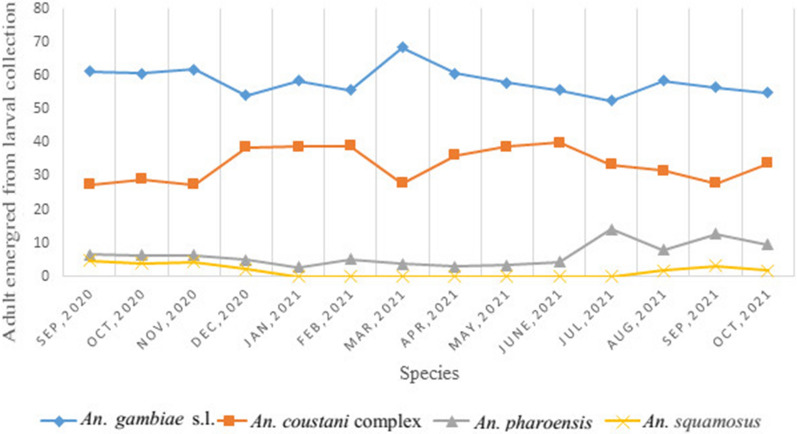
Seasonal abundance of *Anopheles* mosquito species in Arjo-Didessa sugar cane plantation, Ethiopia

As indicated in Table 1 below, *An. gambiae* s.l. had a higher abundance across all three seasons, while *An. coustani* complex and *An. pharoensis* had lower abundances. In this finding, there was a significant number of *Anopheles* species emerging from man-made pools, rain pools, animal hoof prints and earth-bottom irrigation canals throughout the season (F = 4.9632, p = 0.027) (Fig. 4).

**Table 1:** Seasonal *Anopheles* abundance at Arjio Dedessa sugar cane irrigation scheme, Ethiopia

Species	Long rain season (JUN–SEP)	Dry season (OCT–JAN)	Short rain season (FEB–MAY)
*An. gambiae* s.l.	1524 (56.4%)	1959 (58.1%)	714 (59.4%)
*An. coustani* complex	859 (31.8%)	1086 (32%)	443 (36.8%)
*An. pharoensis*	271 (10%)	228 (6.8%)	46 (3.8%)
*An. squamosus*	49 (1.8%)	99 (2.9%)	0
Total	2703 (100%)	3372 (100%)	1203 (100%)

### Molecular identification of the *An. gambiae* complex

**Fig. 5:**
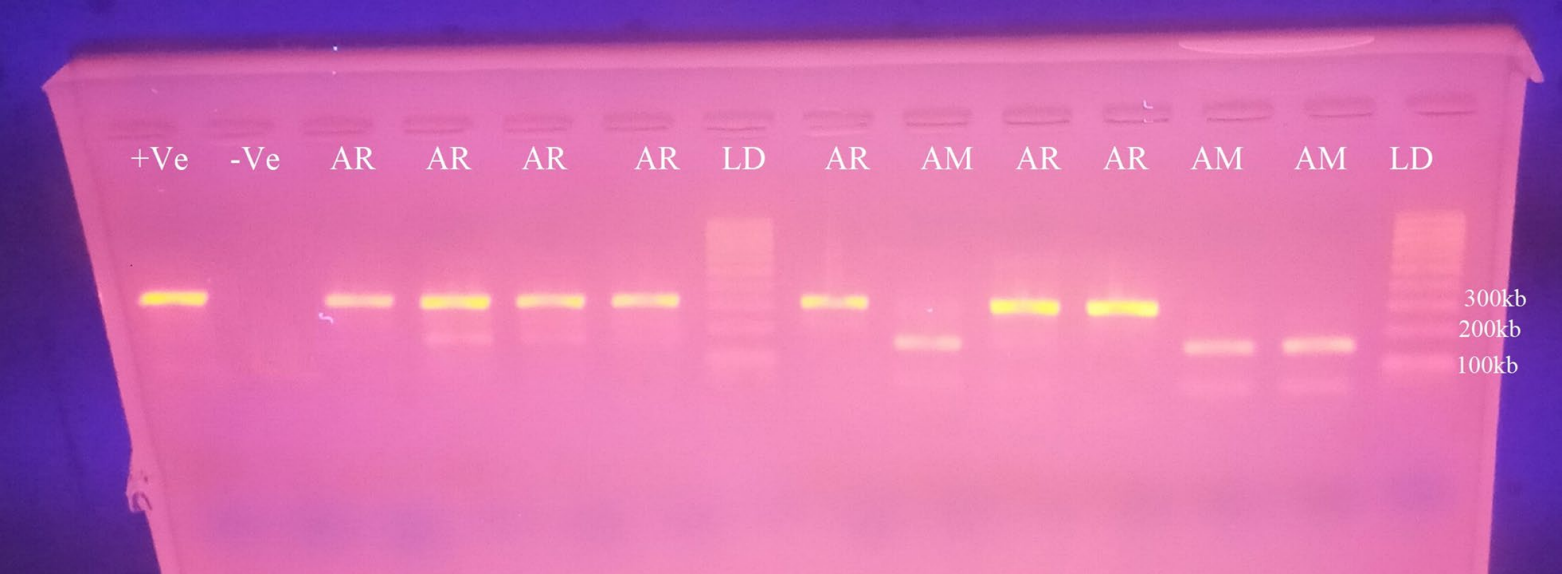
PCR gel electrophoresis result: lanes AR: (315 kb) are *An. arabiensis*; lane AM: (153 kb) *An. amharicus*, lanes LD: 100 kb DNA ladders; lane +VE: + positive control; lane VE: negative control

Out of the 629 *An. gambiae* s.l. sub-samples that were tested for species identification using PCR, 77.9% (n = 490) were found to be *An. arabiensis*, 21.5% (n = 132) were *An. amharicus* (formerly called *An. quadriannulatus* B), and 1.1% (n = 7) could not be amplified (Fig. 5).

### Association between larval abundance and habitat variables

In this study, the regression model depicted that parameters such as season (t = − 2.876, p = 0.00463), distance to the nearest house (t = − 3.847, p = 0.000177), and exposure to sunlight (t = 2.803, P = 005748) were the main predictors of *Anopheles* larval abundance in the study area, whereas habitat type (t = 0.071, p = 0.943466) and habitat permanence were not significantly associated with larval abundance (t = 0.477, p = 0.633859) (Table 2).

**Table 2:** Multivariate regression analysis for assessment of predictors of mosquito larval abundance in presence of different environmental parameters in habitats at Arjo Didessa, sugar cane plantation, Ethiopia

Variable	Estimate	Std. error	t -value	p-value
Physical characteristics				
Types of habitats	− 0.15	2.07	− 0.07	0.943
Season	18.61	6.47	− 2.87	0.004**
Habitat permanence	− 7.49	15.70	− 0.47	0.633
Distance to nearest house	− 26.10	6.78	− 3.84	0.0001***
Exposure to sunlit	28.65	10.22	2.80	0.005**
Physical-chemical characteristics				
Temperature	− 4.90	2.20	− 2.22	0.027*
pH	− 0.15	− 7.20	− 0.02	0.982
Conductivity	1.04	0.32	3.21	0.001**
TDS	− 0.70	− 0.52	− 1.33	0.185
Dissolved oxygen	25.20	3.16	7.96	0.000***
Nitrate ion (mg/l)	276.46	110	2.51	0.013*
Orthophosphate ion (mg/l)	− 14.06	8.64	− 1.62	0.105
Ammonium ion (mg/l)	749.04	331.86	2.25	0.025*

*Significant at p = 0.01, **significant at p = 0. 001 and ***significant at p = 0.0001

Parameters such as temperature (t = − 2.224, p = 0.027), conductivity (t = 3.210, p = 0.001), dissolved oxygen (t = 7.964, p = 4.44e−13), nitrate ion (t = 2.513, p = 0.01305), and ammonium ion (t = 2.257, p = 0.025) were significantly associated with larval abundance. In contrast, pH (0.022, p = 0.98246), TDS (t = − 1.33, p = 0.185), and orthophosphate ion (mg/l) (t = − 1.627, p = 0.105) were not significantly associated with mosquito larval abundance.

*Anopheles* larvae abundance was positively correlated with exposure to sunlight (r = 0.34, p = 0.001), conductivity (r = 0.28, p = 0.001), TDS (r = 0.27, p = 0.001), dissolved oxygen (r = 0.48, p = 0.0010), and nitrate ion (mg/l) (r = 0.37, p = 0.001). Whereas season (r = − 0.22, p = 0.01), types of habitat (r = − 0.20, p = 0.01), and distance to the nearest house (r = − 0.42, p = 0.001) were negatively correlated with *Anopheles* larval abundance (Table 3).

**Table 3:** Correlation of habitat characteristics with *Anopheles* larval abundance

Variables	Mean ± SD	Correlation coefficient	p-value
Types of habitats	6.00 ± 3.17	− 0.20	0.01
Season	1.93 ± 0.80	− 0.22	0.01
Distance to nearest house	2.82 ± 1.12	− 0.42	0.001
Exposure to sunlit	2.55 ± 0.66	0.34	0.001
Conductivity	134.40 ± 52.38	0.28	0.001
TDS	67.82 ± 32.55	0.27	0.001
Dissolved oxygen	6.37 ± 1.51	0.48	0.001
Nitrate ion (mg/l)	0.38 ± 0.06	0.37	0.001

Table 4 indicates that season (t = − 2.228, p = 0.027), distance to the nearest house (t = − 3.812, p = 0.000), exposure to sunlight (t = 2.738, p = 0.006942), and dissolved oxygen (t = − 3.177, p = 0.002) had a significant associated with adult mosquito abundance. However, types of habitats (t = − 0.071, p = 0.943), habitat permanence (t = − 0.477, p = 0.633), and the rest of the physico-chemical variables were not significantly associated with adult abundance (Table 4).

**Table 4:** Multivariate regression analysis for assessment of main predictors of adult mosquito productivity

Variable	Estimate	Std. error	t-value	p-value
Physical characteristics				
Types of habitats	0.29	1.44	0.19	0.842
Season	− 9.98	− 4.48	− 2.22	0.027*
Habitat permanence	− 6.74	10.87	− 0.62	0.536
Distance to nearest house	17.89	4.69	− 3.81	0.000***
Exposure to sunlit	19.37	7.07	2.73	0.006**
Physicochemical characteristics				
Temperature	− 4.18	− 2.28	− 1.82	0.069
PH	6.14	9.88	0.62	0.535
Conductivity	− 0.83	0.47	− 1.77	0.078
TDS	1.59	0.81	1.94	0.054
Dissolved oxygen	− 24.77	7.79	− 3.17	0.001**
Nitrate ion (mg/l)	− 119.68	120.94	− 0.99	− 0.324
Orthophosphate ion (mg/l)	10.02	9.71	1.03	0.303
Ammonium ion (mg/l)	− 158.63	326.46	− 0.48	0.627

**Significant at p < 0.05

Adult *Anopheles* species abundance was positively correlated with habitat permanence (r = 0.28, p = 0.00), exposure to sunlight (r = 0.32, p = 0.00), conductivity (r = 0.28, p = 0.00), TDS (r = 0.27, p = 0.00), and nitrate ion (mg/l) (r = 0.35, p = 0.00). Whereas season (r = − 0.16, p = 0.05), types of habitats (r = − 0.19, p = 0.02), distance to nearest house (r = − 0.41, p = 0.00), and dissolved oxygen (r = − 0.28, p = 0.00) were negatively correlated with adult *Anopheles* mosquito abundance.

## Discussion

This study revealed that larval abundance varies across seasons in different larval habitats. Early dry seasons had a higher larval abundance, whereas long rainy seasons had lower larval productivity. This result was consistent with studies conducted in different parts of sub-Saharan Africa [5, 43–45], which showed that larval abundance and adult productivity decreased in long rainy seasons while increasing in early dry seasons. The reduced larval abundance during long rainy seasons might be the result of an over-flooding effect, i.e., washing of eggs, larvae, and pupa from larval habitats, which was similar to studies conducted in western Kenya and elsewhere in Ethiopia [14, 43, 46].

The high larval abundance in this study correlates with the country’s high malaria transmission season. The main transmission season lasts from October to December in most parts of the country, following the main rainy season from June to September, and there is also a short transmission season from April to May, following the short rainy season [9].

*Anopheles* larvae breed in various types of habitats, ranging from large, permanent collections to small, temporary ones. Irrespective of land setup and types of study, numerous habitat types were identified as breeding sites in this and other studies in Ethiopia [15, 46, 47].

In the long rainy season, in this study, rain pools, animal hoof prints, tire tracks/road puddles, and man-made pools all contributed to a higher proportion of larval abundance. A similar cross-sectional study conducted in the same country indicates that rain pools, animal hoof prints, tire tracks/road puddles were the major contributors during the long rainy season [15, 47]. In contrast, during the long rainy season, river edges, hippo trenches, and spring seepage make no or little contribution. During the dry season, man-made pools, gate valve leakages, earth-bottom irrigation canals, river edges, hippo trenches, spring seepage, and swamps play a greater role in this study. Studies conducted in western Kenya found that larval source management is effective where mosquito larval habitats are accessible and distinct [16]. This would give an opportunity to utilize the seasonality of habitats and larval abundance for the effective suppression of larvae and adult mosquito abundance by targeting larval habitats based on seasonal occurrence.

Studies indicated that there were more than 47 documented species and subspecies of *Anopheles* mosquitoes in Ethiopia [48, 49]. In the current study area, the *Anopheles* mosquito species occurring in the area were *An. arabiensis*, *An. amharicus*, *An. coustani* complex, *An. pharoensis*, and *An. squamosus* [13, 20, 50]. Different findings indicate that in East Africa, the abundance of *An. *gambiae sensu stricto (s.s.) and *An. arabiensis* increased in the early dry season [43–45]. Similarly, in the current study, *An. arabiensis* is highly abundant during the dry season. The rest of the secondary and suspected vectors have a relatively equal proportion. In particular, *An. coustani* complex, regardless of habitat preference, shows a similar pattern of occurrence to *An. gambiae* s.l. in all seasons.

Over the study period, the Simpson model demonstrated that there was species dominance in early, dry, and long rainy seasons by *An. gambiae* s.l. over the other species. Whereas there was species heterogeneity in the late dry season and short rainy season. A similar study conducted elsewhere in East Africa indicates that *An. gambiae* s.l. is the predominant species [43]. In this study, low species diversity was found between larval habitat types. Similarly, studies conducted elsewhere in Africa indicate habitat types have low species diversity [51, 52]. In the current study, species evenness was found to be unequal across seasons and habitats. This contrasts with a study done elsewhere in Ethiopia, which shows that the species were equally distributed with equitability values which indicating the absence of any dominant species [51]. This might suggest that land use practices have significant influence on mosquito species diversity and abundance.

The distance between larval habitat and houses showed an influence on larval abundance in this study, which might indicate the potential for increased indoor vector density [15, 43, 53]. As an evolutionary strategy for energy conservation, it might be suggested that gravid mosquitoes prefer to lay eggs in habitats near human dwellings to conserve energy lost while flying long distances in search of oviposition sites [54].

In this study, water temperature was associated with larval abundance. Similar findings show that larval habitat water temperature is the most important water quality parameter; it affects the quantity of oxygen that can be found in water as a result of photosynthesis by algae and other aquatic plants. Studies reported that moderately high temperatures were necessary for the optimum growth of *Anopheles* larvae [55]. Further, studies indicate elevated water temperatures are also permissible for more microorganisms to grow and serve as a food source for mosquito larvae [56, 57].

The quantity of sunlight significantly affects *Anopheles* larval density. In this study, there was a strong correlation between sunlit exposure and the number of larvae. This finding is similar to previous studies conducted in different sub-Saharan African countries [58–60]. The average daily water temperature is higher in habitats that are directly exposed to sunlight than in habitats that are shaded. The larval growth stage can be prolonged in shaded habitats, which increasing the chance of stunted larvae and the risk of predation [4, 60, 61].

Furthermore, in this study, various physicochemical characteristics, such as conductivity, dissolving oxygen, ammoniums, and nitrate, were found to significantly influence *Anopheles* larval abundances. Recent studies in Ethiopia and neighboring Kenya have found that the above physicochemical characteristics influence *Anopheles* larval abundance [25, 46, 47, 62, 63]. Application of nitrogenous fertilizers in different agro-ecosystems has been demonstrated to lower water turbidity and consequently significantly influence mosquito larval abundance [6, 62–64], since *Anopheles* mosquitoes prefer to oviposit in areas with lower turbidity [65]. In this study, TDS and orthophosphate ions did not show any significant influence on larval abundance. Similarly, phosphate and TDS appear to have no effect on *Anopheles* larval density in a study conducted in western Kenya and Nigeria [21, 66].

Physicochemical studies at different irrigation sites show that agrochemicals, such as herbicides, pesticides, and fertilizers, improve productivity, increase yield, which alter the physicochemical characteristics of larval habitats. This could expose immature stages to different chemicals with similar insecticide groups [27–29]. Selection pressure can lead to the development of insecticide resistance [28, 29, 67]. This could eventually lead to the development of insecticide resistance in malaria vector populations [30, 67]. In different parts of Africa, evidence suggests that agrochemical use results in increases in insecticide resistance in *An. gambiae* s.l. in Burkina Faso and northern Benin [27, 29]. Resistance to *An. arabiensis* in Khartoum State, Sudan, and northern Tanzania [68].

## Conclusions

The study revealed that different larval habitats have different productivity in different seasons. Physical and physicochemical properties such as ammonium and nitrate, as well as the distance between larval habitats and houses, are associated with larval productivity. Therefore, vector control should consider *Anopheles* larval habitat seasonality as well as the impact of agrochemical application on LSM. Furthermore, the current vector control should take into account LSM in the dry season based on seasonal habitat availability in malaria-endemic settings, which may allow for addressing dry-season refugal-larval habitats.

### Supplementary Information

**Additional file 1.** Mean larval density by breeding habitat type, Arjo Didessa sugar cane plantation, Ethiopia. **Additional file 1.**

## Data Availability

The corresponding author can provide the datasets used in this study upon reasonable request.

## Abbreviations

LSM: Larval source management
SDI: Species diversity index
TDS: Total dissolved solids
