# Supplementary figures and images for: Anopheles larval habitats seasonality and environmental factors affecting larval abundance and distribution in Arjo-Didessa sugar cane plantation, Ethiopia
Source: Malar J. 2023 Nov 15;22:350. doi: 10.1186/s12936-023-04782-1 (PMC10652594; doi:10.1186/s12936-023-04782-1)

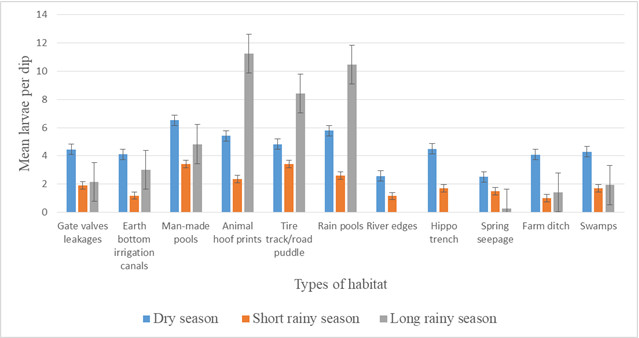

Supplement: Supplementary file 1 — Additional file 1. Mean larval density by breeding habitat type, Arjo Didessa sugar cane plantation, Ethiopia. [file 12936_2023_4782_MOESM1_ESM.jpg]
